# Zinc Therapy in Early Alzheimer’s Disease: Safety and Potential Therapeutic Efficacy

**DOI:** 10.3390/biom10081164

**Published:** 2020-08-09

**Authors:** Rosanna Squitti, Amit Pal, Mario Picozza, Abofazl Avan, Mariacarla Ventriglia, Mauro C. Rongioletti, Tjaard Hoogenraad

**Affiliations:** 1 Molecular Markers Laboratory, IRCCS Istituto Centro San Giovanni di Dio Fatebenefratelli, 25125 Brescia, Italy; 2 Department of Biochemistry, All India Institute of Medical Sciences (AIIMS), Kalyani, Nadia, West Bengal 741245, India; maximus1134@gmail.com; 3 Neuroimmunology Unit, IRCSS Fondazione Santa Lucia, 00143 Rome, Italy; m.picozza@hsantalucia.it; 4 Department of Public Health, Mashhad University of Medical Sciences, Mashhad 91778-99191, Iran; abolfazl.avan@gmail.com; 5 Fatebenefratelli Foundation for Health Research and Education, AFaR Division, 00186 Rome, Italy; mariacarla.ventriglia@afar.it; 6 Department of Laboratory Medicine, Research and Development Division, San Giovanni Calibita Fatebenefratelli Hospital, Isola Tiberina, 00186 Rome, Italy; maurociroantonio.rongioletti@fbf-isola.it; 7 Retired neurologist at the Department of Neurology, University Medical Centre, 3584 CX Utrecht, The Netherlands; tjaard.hoogenraad@gmail.com

**Keywords:** zinc therapy, mild cognitive impairment, Alzheimer’s disease, Wilson disease safety, efficacy, copper

## Abstract

Zinc therapy is normally utilized for treatment of Wilson disease (WD), an inherited condition that is characterized by increased levels of non-ceruloplasmin bound (‘free’) copper in serum and urine. A subset of patients with Alzheimer’s disease (AD) or its prodromal form, known as Mild Cognitive Impairment (MCI), fail to maintain a normal copper metabolic balance and exhibit higher than normal values of non-ceruloplasmin copper. Zinc’s action mechanism involves the induction of intestinal cell metallothionein, which blocks copper absorption from the intestinal tract, thus restoring physiological levels of non-ceruloplasmin copper in the body. On this basis, it is employed in WD. Zinc therapy has shown potential beneficial effects in preliminary AD clinical trials, even though the studies have missed their primary endpoints, since they have study design and other important weaknesses. Nevertheless, in the studied AD patients, zinc effectively decreased non-ceruloplasmin copper levels and showed potential for improved cognitive performances with no major side effects. This review discusses zinc therapy safety and the potential therapeutic effects that might be expected on a subset of individuals showing both cognitive complaints and signs of copper imbalance.

## 1. Introduction

Zinc has been utilized for some time now in the treatment of Wilson disease (WD), an autosomal rare disease of copper imbalance, which mainly harms brain and liver, caused by mutations in the *ATP7B* gene. WD is diagnosed on the basis of several symptoms and biomarkers that encompass hepatic, neurological, or psychiatric clinical signs, low levels of ceruloplasmin, increased levels of non-ceruloplasmin bound copper (also known as ‘free’ copper) in serum and urine, and the identification of *ATP7B* gene mutations [1,2]. 

Alzheimer’s disease (AD) is the most common form of dementia in the elderly. It exists in two forms: ‘familial’ and ‘sporadic’. Familial AD (fAD) is rare, but it has an early onset and a known causative mutation in amyloid precursor protein (APP), presenilin (PSEN) 1 or PSEN2 genes, inherited as an autosomal dominant trait. Mutations in these genes affect the normal processing of APP and increase the brain levels of amyloid-beta 1-42 (Aβ) believed to be the main factor in the pathological basis of the disorder [3]. Sporadic AD is a heterogeneous complex disorder with a late onset (over 65 years). A subset of patients with AD and its prodromal form, known as Mild Cognitive Impairment (MCI), fail to maintain a normal copper balance and distribution in the body. Despite a wealth of knowledge on AD mechanisms, current treatments fail to show full efficacy.

Herein, we discuss the applicability of a zinc-based approach to restore copper balance in a subset of MCI patients exhibiting non-ceruloplasmin bound copper values higher than normal (0.1–1.6 µmol/L [4]), by using zinc treatment in WD as a template.

### 1.1. Non-ceruloplasmin Copper Typifies a Subset of Alzheimer’s Disease Patients

Alzheimer’s Disease is characterized by an aberrant misfolding and accumulation of beta-amyloid (Aβ) protein that forms extracellular deposits in the brain [5,6] and by abnormal phosphorylation of the microtubule associated protein tau that forms intraneuronal aggregates of neurofibrillary tangles [7]. Despite the massive knowledge about the Aβ cascade mechanisms, its exact role in AD pathology, along with the genetic and environmental factors that modify the onset and course of the disease, remains not fully understood. Besides the most popular AD hypothesis, the Amyloid Hypothesis [5], questioned in the latest years [8], the Metal Hypothesis [9] postulates that the interaction between iron, copper, zinc, and AD-related proteins (encompassing the amyloid precursor protein (APP)/Aβ system and tau proteins) are important drivers of AD pathology [8,10]. This hypothesis [9], postulated some years ago, proposes that the loss of metal balance in distinct brain areas can alter the redox state, trigger mitochondrial and autophagic dysfunctions, and set in motion some neurodegenerative processes (reviewed in [11]).

Existing meta-analyses and epidemiological studies sustain the Metal Hypothesis and point to copper imbalance as an important contributor to AD susceptibility (reviewed in [11,12]). In fact, increasing evidence shows that a subset of AD/MCI individuals is characterized by higher than normal levels of non-ceruloplasmin copper, and it is analogous in the brain known as labile copper [13,14], resembling WD [15]. While copper is essential for the activity of many enzymes and proteins in correct physiology ensuring brain development and functioning, its loss from copper-dependent enzymes and copper-transporting proteins facilitates the expansion of the pool of non-ceruloplasmin copper. Copper from this pool crosses the blood-brain barrier (BBB), fueling labile copper in the brain that engages in toxic Fenton reactions that produce very reactive radicals, threatening neuronal physiology. Readers can refer to recent reviews [11,12] for an updated discussion of the pleiotropic toxic effects of copper imbalance in AD and its implication in brain antioxidant stress, abnormal Aβ, and tau misfolding/aggregation, as well as aberrant mitochondrial function and energy production postulated at the basis of AD neurodegeneration. 

Some authors speculated that: ‘…there are two neurodegenerative diseases with abnormalities in copper metabolism: (a) the juvenile form with degeneration in the basal ganglia (WD) and (b) the age related form with cortical neurodegeneration (AD)…’ [16]. This opinion is now supported by massive evidence (reviewed in [12]). As in WD, zinc treatment might be beneficial in improving all of the aberrant processes at the basis of AD-copper toxicity.

### 1.2. Non-ceruloplasmin Copper, Astrocytes and Alzheimer’s Disease Axis

Astrocytes, the copper depots of the brain [17], play a paramount role in homeostasis of the redox-active metal copper, which, upon dyshomeostasis, is detrimental to cell functioning and survival [12]. Several lines of evidence have demonstrated the central role of reactive astrocytes in the early cellular phase of AD (reviewed in [18]), even before the appearance of Aβ deposits, suggesting it as a very early event. Strikingly, these cells are also closely involved in Aβ catabolism [19]. 

Reactive astrocyte is a dual edge sword. The beneficial effects of astrogliosis are mediated through scar formation, which helps in holding the spread of inflammatory cells [20]. On the other hand, reactive astrogliosis exerts neurotoxic effects by facilitating increased levels of inflammatory cytokines (reviewed in [21]).

Labile trace elements concentrations in different brain regions, otherwise meticulously maintained within narrow limits, are deregulated upon aging and inflammation at the choroid plexus due to perturbed BBB [22], and further increase during AD course (reviewed in [11,12]). Astrocytes and microglia both confer protection by scavenging metals that cross BBB, thereby protecting neurons by attenuating neural excitotoxicity [23]. Clinical studies have shown that around 3% of non-ceruloplasmin copper can cross the BBB as its filterable [14]. If non-ceruloplasmin copper levels keep on rising unchecked, it might be expected that the protective reversible reactive astrogliosis event may result in irreversible neuroinflammatory response, which can be detrimental for neuronal health. Neuroscience that is related to Copper-Astrocytes-AD axis is still at an infancy stage, setting a perfect stage for cutting edge basic, experimental and clinical research for further exploration. 

Taking into account the strong assistance that exists between neurons and astrocytes [24] alongside pivotal role of astrocytes in CNS metal homeostasis, it can be opined that aberrant astrocyte biology leads to the deprivation of ideal microenvironment around neurons, which can have direct injurious consequences to brain parenchymal cells. One would optimistically envisage that lowering the levels of the labile copper through the reduction of its analogues non-ceruloplasmin copper in the bloodstream by means of zinc therapy might improve the AD outcome or even halt the disease progression (in preclinical stage and/or prodromal MCI subjects) also due to improved astrocytes functioning, which needs to be clinically tested. However, the role of reactive astrogliosis in response to metal toxicity is enigmatic, and is further compounded by large heterogeneity in astrocyte morphology and physiology [24]. Additionally, there is not enough clarity over whether reactive astrogliosis is the cause or consequence of excess labile copper toxicity. The accumulation of labile copper may be downstream compared to other damaging factors and consequent to significant dysfunction of the BBB. 

## 2. The Zinc-copper Connection in Microglial Cell Function and Alzheimer’s Disease

Among immune cells, the brain is primarily populated by resident perivascular macrophages and microglia. Apolipoprotein E (APOE) is the strongest sporadic AD risk gene [25]. APOE binding to Aβ promotes its clearance by microglia, whereas the AD-predisposing *APOE4* allele is associated with the impairment of this process [26]. 

*APOE4* gene-replaced mouse macrophages show impaired efferocytosis, the process by which phagocytes engulf and eliminate apoptotic cells [27]. Reduced intracellular zinc levels may aggravate this genetically driven dysfunction [28], arguing for beneficial effects of early zinc therapy in *APOE4* carriers by enhancing the scavenger functions of microglial cells and of perivascular macrophages. 

Neuroinflammation does not only depend on the accumulation of copper in the brain and, on the contrary, it can probably precede the metal accumulation, which is therefore downstream. 

However, the Cu-lowering action of zinc supplementation may be also exploited to regulate brain inflammation in AD. Divalent zinc cations are known to induce conformational changes in fibrin and formation of complexes with heparin and thrombin [29], but the effect of zinc on pro-inflammatory pathways that are mediated by CNS fibrin, as well as on the affinity of fibrin for Aβ are still not known and would likely have interesting implications for AD management and prevention.

### 2.1. Zinc in Physiology

Zinc is an essential trace element that is required for the function of over 2000 metallo-enzymes/proteins in diverse non-enzymatic biological reactions and serves as a crucial component in the regulation of DNA and RNA synthesis, in hormone-receptor interactions, and as second messenger for intracellular signaling, neurotransmission, neurogenesis, or neuronal growth (reviewed in [12,30]). Zinc deficiency is a worldwide problem and the effects of malnutrition may be relevant for neurodegenerative diseases as the brain is the organ with the highest zinc levels. Dietary zinc supplements usually are prescribed as zinc sulphate or zinc gluconate.

Zinc is absorbed in the small intestine, especially in the jejunum, and it is responsive to dietary intake (reviewed in [31]). When zinc ingestion exceeds the normal dietary levels, intestinal metallothioneins (MT; cysteine-rich intracellular proteins that are capable of binding and sequestering ions) are induced, causing some zinc to be bound in the mucosal cells and presumably being lost as cells slough off. Zinc homeostasis is regulated by Zinc transporters (ZnT) and zinc-regulated and iron-regulated transporter proteins (Zip) solute-linked carriers. ZnT and Zip transporters have opposite roles within the cell, ZnT being mostly involved in decreasing, while Zip transporters in increasing, cytoplasmic zinc concentrations. ZnT1 is located in small intestine and it regulates zinc release from the enterocyte to general circulation. 

**Figure 1:**
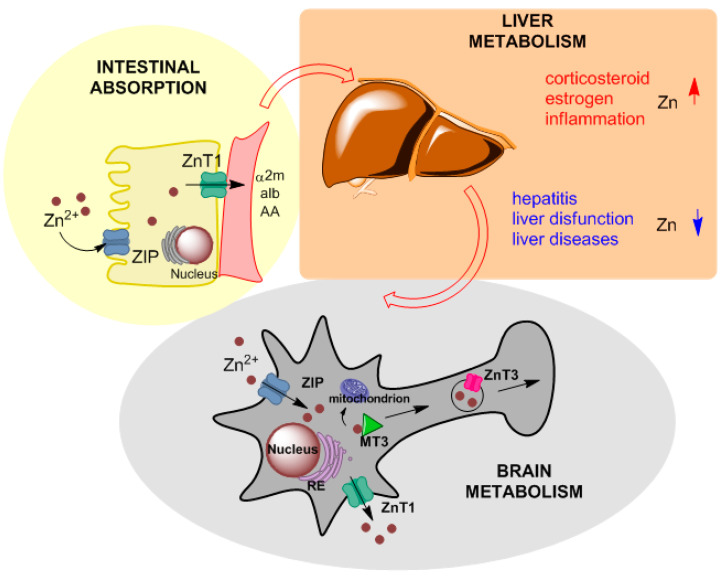
General zinc absorption and metabolism. *Intestine:* zinc is absorbed in the small intestine and is responsive to dietary intake. ZnT and Zip transporters have opposite role within the cell: Zip transporters in increasing cytoplasmic zinc concentrations while ZnT in decreasing it, in particular ZnT1 is located in small intestine and regulates zinc release from the enterocyte to general circulation. Zn^2+^ is tightly bound to α2-macroglobulin (α2m) and loosely to albumin (Alb) and other proteins, peptides and amino acids (AA) which serve as primary sources of zinc accessible to all cells. *Liver:* The liver plays a central role in zinc metabolism as well. Corticosteroids, estrogens and inflammation decrease plasma zinc, associated to an increase of zinc in the liver. Hepatitis, liver dysfunction, or liver diseases with fulminant, but also chronic or subacute hepatic failure are associated with low levels of zinc. Reduced levels of zinc are found in alcoholic hepatitis patients, while they are less decreased in nonalcoholic liver disease. *Brain:* zinc is necessary for brain development and physiology. The Zn^2+^ ions could be transported into neurons by the transmembrane ZiP proteins. Once inside the accumulation and trafficking of Zn^2+^ are directed by members of ZnT family. ZnT1, 3, and 6 are abundant in the brain and central nervous system (CNS). The homeostasis of Zn^2+^ in the brain is also regulated by the MT3 protein. Zinc is sequestered into presynaptic vesicles by ZnT3, which is coupled with vesicular glutamate transporter (not shown); zinc is then released into synaptic cleft together with glutamate (not shown).

Most of body zinc is stored in skeletal muscle, bone, liver, and the brain, while plasma zinc accounts for less than 1% of total zinc and its concentrations are 10–15 µmol/L. It is tightly bound to α2-macroglobulin (30%) and loosely to albumin and other proteins, peptides and amino acids (70%) which serve as primary sources of bioavailable zinc. Some constituents of the diet have an effect on zinc absorption. A diet that is rich in animal proteins results in greater zinc absorption, while phosphorus-rich phytates present in seeds and nuts reduces the absorption of zinc with important effects on its bioavailability [31]. Zinc is mainly excreted through the intestine, in the pancreatic secretions, while urinary loss and shedding of epithelial cells are lower (Figure 1). 

The liver also plays a central role in zinc metabolism. Corticosteroids or estrogens, but also inflammation, have the effect of decreasing plasma zinc, associated to an increase of zinc in the liver. Hepatitis, liver dysfunction or liver diseases with fulminant but also chronic or subacute hepatic failure are associated with low levels of zinc. Lower zinc levels are found in alcoholic hepatitis than in nonalcoholic liver disease patients (Figure 1).

Zinc is not metabolized and its half-life elimination in healthy subjects is in the range 0.9–1.2 h. 70–80% elimination results from fecal excretion, while relatively little is excreted from urine and sweat (15–25%). Following zinc oral administration, urinary excretion is very low (up to 2% of the administered dose), while fecal excretion varies greatly (20–76% of the administered dose). Zinc in the stools is in the greatest part due to the passage of unabsorbed zinc, but also to endogenous intestinal secretion. Bile contains very small zinc, as opposed to pancreatic secretions, which may play a significant role in zinc homeostasis [32].

Zinc homeostasis in the brain is primarily regulated by MT, ZnT, and members of the ZiP family and in vitro increased levels of zinc showed a response of BBB with an increased activity of zinc regulatory proteins that sequester excess zinc into intracellular vesicles [33]. Metallothioneins-3 is exclusively expressed in the CNS and it has reported to partake in sequestering zinc in synaptic vesicles [34]. It is significantly down-regulated in AD [35,36]. It was recently proven that this protein protects cultured neurons from toxicity generated by Aβ. A metal swap between MT-3 and soluble aggregated Aβ 1-40-Cu^2+^ avoids reactive oxidative species production and consequent cellular toxicity.

Although previous and recent studies have highlighted conflicting results on zinc levels in AD patients, existing meta-analyses identified decreased serum zinc in AD patient with respect to healthy elderly controls [37], suggesting the potential role of zinc systemic dyshomeostasis in the pathogenesis of AD [38]. This evidence may be due to nutritional zinc deficiencies and/or deficiency in the expression/activities of zinc regulatory proteins [38]. Some investigations in the brain of AD patients demonstrated that they differed from healthy elderly controls in terms of the expression of zinc regulatory proteins [39,40]. 

Zinc is finely regulated in the body and this limits its potential toxic effects, even at high dosage, particularly within the brain. In this organ, toxicity is triggered when the endogenous zinc fluxes within the cells are disturbed, e.g., after transient global ischemic insults that trigger excitotoxic processes, caused by excessive release of glutamate neurotransmitter at the synapse. Ca^2+^, but also Zn^2+^ concentrations, buildup within the cell during excitotoxicity and prompt neurodegeneration [12], in this case, zinc toxicity could be prevented by the intraventricular injection of zinc-chelating agents [41,42]. The mechanisms by which zinc exerts its toxicity during ischemic insults seems to be ascribable to structural abnormalities in tubulin or microtubule associated proteins assembly [43]. In WD, dosages of zinc in the range of 100–150 mg/day do not exacerbate neurological symptoms. In AD, six month therapy at 75–150 mg/day did the same [44].

Zinc deficiency, rather than excess, is deleterious for many biological functions. Acrodermatitis entoropathica is the only genetic defect resulting in zinc homeostasis disturbance described, and it is treated with zinc therapy. No genetic disease has been reported to increase zinc concentrations or have effects on brain function. Supplementation is beneficial for immune dysfunctions, diarrheal disease, liver, and neurological diseases [31].

### 2.2. Zinc Clinical Information

Zinc’s mechanism of action at dosage of 100–150 mg/day involves the induction of intestinal cell MT, which blocks copper absorption from the intestinal tract and results in a negative copper balance [45]. This negative balance is caused by the blockade of both the absorption of dietary copper and of the reabsorption of copper secreted in saliva, gastric juice and intestinal secretions. Zinc is also a cofactor for polymerases and proteases that are involved in many cellular functions (e.g., wound repair and intestinal epithelial cell regeneration). Furthermore, zinc is a cofactor for thymulin, a thymic hormone that is essential for T-cell maturation. 

Zinc has antioxidant properties and it may protect against macular degeneration from oxidative stress. These properties are at the basis of therapeutic indications of zinc for the treatment of wounds, burns, and acne vulgaris.

Zinc is very effective in controlling toxic non-ceruloplasmin copper levels in WD and other copper toxicity diseases (Table 1). Excess non-ceruloplasmin copper is toxic, since it is involved in deleterious oxidation of lipids and proteins and in free radical formation. Likewise, zinc showed plausible efficacy in normalization of copper levels in AD [44]. The main advantages of zinc over other anti-copper agents is its extremely low toxicity and no iatrogenic copper intoxication [46]. The only side effect is gastric upset in approximately 10% of patients, which usually decreases over time. As with all anti-copper therapies, over a long period of time, overtreatment and induction of copper deficiency can occur [47].

A large double-blinded randomized controlled clinical trial of high-dose zinc alone (80 mg of zinc as zinc oxide) or in combination with antioxidants (500 mg of vitamin C, 400 IU of vitamin E, and 15 mg of beta carotene) and copper (2 mg of copper as cupric oxide) on 3741 participants from 82 academic and community medical centers in the US (the Age-Related Eye Disease Study 2, AREDS) showed a significant reduction in the risk of progression to advanced age-related macular degeneration with no adverse drug reactions (ADRs) [48].

## 3. Zinc Therapy Indications and Dosage

Zinc sulphate has been employed in the therapy of WD since 1961, when Schouwink described the symptoms improvement in two patients that were treated with zinc sulphate in the Netherlands [3,4,5,6]. Subsequently, in 1977, Schouwink’s colleague, Hoogenraad started administrating zinc sulphate to WD patients and published his experience of 27 WD patients in 1987 [49]. Since that time, George Brewer carried out intense research into the use of zinc in treating WD, while using the acetate salt [50].

European Medicines Agency (EMA) has approved zinc sulphate (IDI Farmaceutici S.R.L.) in 2000 for therapy and prophylaxis of zinc deficiency also during pregnancy and breastfeeding and for the treatment of enteropathic acrodermatitis, treatment of wounds and burns, and treatment in acne vulgaris. 

In 1997, based on Dr. Brewer’s data, zinc acetate was approved for WD maintenance therapy in the US by the FDA under the trade name Galzin [32,47,51]. Later, in 2001, zinc acetate was authorized by the EMA under the trade name Wilzin, and marketed by Orphan Europe SARL, France. Following its marketing authorization in the U.S.A., Europe, and later Japan (trade name Nobelzin), zinc quickly became the WD maintenance therapy of choice around the world.

### 3.1. Rational of Zinc Therapy in Alzheimer’s Disease

The global prevalence of dementia has been estimated to 9.9 million new cases worldwide (World Alzheimer report 2015; http://www.alz.co.uk/research/world-report-2015), implying one new case every 3.2 seconds. The 2020 AD facts and figures [52] estimated that 5.8 million Americans of all ages are living with AD in 2020 and the total payments for health care, long-term care, and hospice services for people age > 65 years with dementia are estimated to be $244 billion. The total lifetime cost of care for someone with dementia was estimated at $305 billion dollars in 2020. 

The current consensus in the field of AD is that the cause of AD is incompletely defined, and no truly effective therapy exists. Clinical trials that are based on Aβ destroying enzymes or anti-Aβ compounds have delivered negative results so far [53]. 

However, beneficial results have been provided in early 2020 by a phase III clinical trial with aducanumab (EMERGE; https://www.alzforum.org/), showing improvements either in removing Aβ plaques or in slowing cognitive decline. In the same line, a new drug based on Oligomannate has been approved in China for AD treatment. It has been announced that it will be tested in a phase III clinical trial in China, the US, and Europe to authorize the marketing of the drug in these countries [54]. 

The Interventional study Dominantly Inherited Alzheimer Network (DIAN)-TU was a phase 2/3 clinical trial assessing cognitive efficacy of two monoclonal antibodies against Aβ in individuals with mutations causing dominantly inherited AD. Despite the marked reduction of Aβ plaques achievable by these biologic drugs [55], the study missed the primary endpoint based on cognitive testing in early 2020 [56], suggesting that Aβ accumulation, the pathological hallmark of AD, is not necessarily the unique factor influencing cognitive decline in AD [57]. As a matter to the fact, abnormal amyloid deposition exists in 20–40% of non-demented individuals [58,59]. This points out that AD, and especially the sporadic form of the disease, is a complex and heterogeneous disorder that may coalesce from multiple risk factors that include the deposition of Aβ plaques. Supporting this view, recent studies have implicated metabolic abnormalities in AD pathology. Besides Aβ metabolism abnormalities, insulin resistance, hormonal deficiencies, hyperhomocysteinemia, and copper imbalance [11,12] have been implicated in the pathogenesis of the disease, likely linked to each other and to the APP/Aβ system [12,60]. It is well documented that Aβ is a metallopeptide, and Cu^2+^ and Zn^2+^ change its aggregation state, structure, and toxicity, facilitating metal exchanges, at the synapses in neuron cell membranes [61,62,63,64,65]. Recently, a critical, location-dependent copper dissociation constant (K_d_^c^) has been proposed as a new mechanistic model featuring copper imbalance in AD: it proposes the shift from physiological protein-bound copper ion pools to loose copper toxic pools [11]. According to this model, the AD copper misbalance can be described as the loss of functional copper from protein-bound pools and the build-up of a pool of copper unbound to proteins that gains redox-toxic functions [11]. In the brain, this mechanism can facilitate the formation of Aβ oligomers at the glutamatergic synapse (recently reviewed in [66]), which are the best known and proven toxic factors in AD, and prompt their aggregation to form insoluble plaques and favor the formation of amyloid plaques short-circuiting neuronal networks. Copper can also affect the function and structure of crucial copper proteins, including prion proteins and α-synuclein [11], and facilitate the accumulation of phosphorylated tau within the neurons due to the presence of Cu-binding sites on the protein [67], in line with Metal Hypothesis of AD [9]. 

In the future, we should be able to begin to determine the best potential intervention for individuals who complain of loss of cognitive performances on the basis of their susceptibility to different metabolic alterations associated to the disease. Pursuing the shift from a one-size-fits-all approach (that have still provided negative results) to treatment regimens that are tailored to individual metabolic disorders holds promise for beneficial intervention to slow the progression of the disease based on personalized or precision medicine.

The rationale for zinc therapy phase II clinical trials is based on the critical need for disease-modifying AD treatment and prevention and on the concept that zinc therapy represents a potentially beneficial intervention to delay disease progression for a subset of MCI/AD patients, who exhibit susceptibility for copper dyshomeostasis. Zinc is a copper competitor in intestinal absorption, potentiating the MT block at the intestinal level (‘mucosal block’), consisting in a 25-fold increase of MT expression, which traps copper into enterocytes for excretion [45]. During therapy with a dosage of 100–150 mg/day of elemental zinc, body copper balance becomes negative, reverting copper compartmentalization and distribution from the blood to organs and tissues, including the brain [45]. 

The treatment has the ultimate goal of counteracting oxidative stress, aberrant mitochondrial function, and amyloid dysregulation by mobilizing copper from Aβ plaques, so as to promote the redistribution of the metal from the brain to the bloodstream. 

Other added values of this approach are based on the fact that zinc supplementation is a powerful driver for neurotrophic signaling and neuronal plasticity that is mediated by increased levels of the Brain-derived neurotrophic factor (BDNF) [38] and that AD patients suffer from slight zinc deficiency, as discussed before [37,68].

### 3.2. Early Studies on Zinc Therapy in Alzheimer’s Disease

Initial studies of zinc therapy in AD patients were originally undertaken by Constantinidis [69,70,71] and also later suggested by Burnet [72]. Constantinidis (1992) [71] postulated that a non-specified genetically determined disorder of the BBB, depending on microvascular neuropile-capillary amyloid plaques, would have been responsible for an influx of metals (calcium, iron, aluminum, silicon, mercury, copper, etc.) into the brain cortex. In turn, such an increase of metals in the brain would have displaced zinc, untimely leading to zinc deficiency in the AD brain, especially detectable at the level of the hippocampus, one of the brain sites normally enriched in this element. A recent review [73] summarizes findings on the potential efficacy of zinc therapy for prevention and the improvement of cognitive decline. However, these studies carried out on AD patients had small sample sizes (Table 2). For example, a study evaluating 10 individuals with AD treated for three to twelve months with 150 mg of elemental zinc daily showed an improvement in memory; for eight patients, improvement of memory, understanding, communication, and social contact were evident [71]. Another study carried out on four individuals suggested modest improvements on Mini-Mental State Examination (MMSE) after three months [74]. A previous prospective, randomized, six-month double-blinded, parallel, placebo-controlled phase II study on 46 AD patients from Brewer’s group [44] missed its primary endpoint, but demonstrated that zinc effectively lowered levels of non-ceruloplasmin copper in AD patients, resuming correct physiology [44] and improved cognitive scores in ADAS-Cog (*p* = 0.037) and CDR SOB (*p* = 0.032) and MMSE (*p* = 0.07)] in post-hoc analysis, including only patients who were 70 + years old (14 zinc treated patients vs. 15 placebo). A consistent percentage of treated patients were also cognitively stabilized six months after the study completion (Brewer personal communication). Overall, these studies have obvious weaknesses, mostly in the study design and inclusion criteria, but they suggest potential beneficial effects in terms of improved cognitive performances and control of copper dyshomeostasis and do not report major side effects in patients with cognitive deficits or AD.

They were primarily hampered by a small sample size and the limited patient stratification as far as the copper and AD status is concerned. All of these issues and shortcomings can be easily overcome by maximizing the possibility of early intervention recruiting individuals diagnosed as MCI [83,84,85,86,87,88,89,90] and employing non-ceruloplasmin copper as a serum stratification/prognostic biomarker [91,92] and inclusion criterion (higher than 1.6 µmol/L) for eligibility assessment to participate in clinical trials testing zinc therapy.

On this basis, we planned a 24-month, multicentre, randomised, double-blind, placebo-controlled, parallel group, phase II clinical trial in order to evaluate the efficacy and safety of Zinco Solfato (IDI Farmaceutici) in patients with MCI (EudraCT 2019-000604-15). We will evaluate 215 MCI patients with non-ceruloplasmin copper >1.6 μmol/L (normal reference range 0.1–1.6 μmol/L; [4]) and positive for AD related cerebrospinal fluid biomarkers (peptides Aβ, tau, p-tau) or for the presence of Cerebral Amyloidosis evaluated by Florbetapir (18-F)-Positron emission tomography.

### 3.3. Zinc Therapy: Dosage, Adverse Drug Reactions and Preventive Measures 

Employing tablets of 50 mg of elemental zinc, the zinc dosage used in WD treatment (2–3 tablets/day), zinc deficiency during pregnancy and breastfeeding (1–2 tablets/day), enteropathic acrodermatitis (10 mg/Kg/day), wounds and burns (2–3 tablets/day), and acne vulgaris (2–4 tablets/day) might suggest the suitable dosage to employ for MCI. On this basis, in early clinical trials testing zinc supplementation in MCI, the appropriate dosage of zinc might be 2–3 tablets/day [45]. In individuals with MCI exhibiting higher than normal values of non-ceruloplasmin, this dosage might cause possible ADRs, as follows: sideroblastic anemia (uncommon) and increased levels of serum amylase, lipase and alkaline phosphatase (common). Członkowska & Litwin [93] reported that zinc sulphate seems to cause fewer severe ADR than chelating agents; most frequently gastritis (stomach pain) or an increased level of pancreatic enzymes (without clinical signs) (reviewed in [93]). Of 72 patients with WD, who started treatment with zinc sulphate, only two (2.7%) needed a change in treatment, because of ADRs (i.e., skin reaction and thrombocytopenia) (reviewed in [93]). These authors noted that patients who experienced stomach pain should take the medication half an hour after their meal, but not on an empty stomach. There is no study comparing the ADRs of different zinc salt formulations.

The prolonged administration of high-dose zinc compounds can lead to copper deficiency (hypocupremia). Copper deficiency can induce anemia that is recovered rapidly following the reduction of zinc dosage and careful hematological and ceruloplasmin monitoring can help to identify copper deficiency since early manifestations. 

Serum ceruloplasmin is regarded as a surrogate marker of copper status in non-Wilson patients [94]. Ceruloplasmin is synthesized in the liver and secreted into the blood at a rate that is dependent upon the availability of copper to the liver [95]. As the copper levels decrease, the secretion of ceruloplasmin decreases; thus, the serum ceruloplasmin level is a good surrogate measure of the copper availability for biologic use. The use of ceruloplasmin as a surrogate marker of copper arises from animal model studies and, subsequently, has been used in clinical studies on patients, other than WD [94]. A low ceruloplasmin level can indicate copper deficiency before anemia occurs. 

The initial indication of zinc overtreatment often is mild anemia and/or leukopenia, which is reversible by lowering the zinc dose, or if more severe, temporarily stopping drug administration. This is rarely seen at ceruloplasmin levels over 10 mg/dL, but it is more frequent at ceruloplasmin levels between 5 and 10 mg/dL, and is much more common below 5 mg/dL. Thus, in early clinical trial based on zinc therapy, zinc should not be prescribed in case of a ceruloplasmin level lower than 10 mg/dL. Furthermore, during the initial period ceruloplasmin and blood counts should be assessed at weeks three, six, and 12 to monitor zinc overtreatment. If there is a 20% or more drop in hemoglobin, white blood cell, or platelets, or ceruloplasmin values below 10 mg/dL, zinc administration should be temporarily halted until the count recovers, and then resumed at 50% of the patients previously administered. If there is a recurrence, a drug holiday is recommended, until neither mild anemia nor leukopenia will be present, as assessed by blood counts, with the lowest dose admitted equal to one elemental zinc tablet of 50 mg/day.

Another common ADR of zinc therapy in WD is gastric irritation and it derives from the fact that it has to be taken three times daily on an empty stomach to be efficacious (two hours far from meals). Additionally, in this case, zinc administration should be halted until recovery and then resumed at 50% of the patient’s previously administered dosage, as described above. Non-ceruloplasmin copper quantification can be employed in early phase II zinc therapy clinical trials in MCI also as a drug efficacy biomarker. During zinc therapy, the measurement of serum non-ceruloplasmin copper, which should approach normal range (lower than 1.6 µmol/L [4]), will ensure the biochemical efficacy of the treatment.

Since early ’90, it became clear that soluble Aβ, reacting with zinc and copper, precipitates as insoluble aggregates in vitro [96]. On this basis, it has been proposed that a breakdown of zinc homeostasis could play a role in AD pathology [97,98]. More specifically, it has been proposed that an aberrant zinc homeostasis might cause endogenous excess of zinc in the neuronal cells, in the form of floods released from the cell during synaptic transmission or as a consequence of perfusion-reperfusion states induced by head trauma [99,100]. However, oral zinc administration has not been shown to affect intraneuronally zinc fluxes or faliticitate aberrant zinc hoemostasis apart from transient global ischemic insults. 

## 4. Conclusions

When considering the dosage zinc has only minor gastric intolerance in a number of patients with no long-term side effects. Furthermore, during zinc therapy, different biomarkers will regularly be checked to monitor drug safety and efficacy. Being a natural substance, zinc is not subject to the risk of long-term immune reactions. Thus, because of its safety profile and high efficacy in the treatment of WD, zinc therapy is worth studying as a therapeutic option for MCI individuals with abnormal non-ceruloplasmin copper levels. If successful, this potential therapeutic option will also take advantage of the availability of zinc drugs already in the market at very low cost with beneficial effects in terms of timing of drug availability for the public and limited costs for Health Care Systems.

## Figures and Tables

**Table 1:** Summary of diagnosis and management of patients with copper diseases.

Manifestations	Movement disorder, liver disease, low ceruloplasmin (<200 mg/L), high serum non-ceruloplasmin copper (>100 μg/L),* increased urinary copper (>100 μg/L), Kayser-Fleischer ring
Cause of symptoms	Increased non-ceruloplasmin bound (free) copper * (>100 μg/L)
Aim of medication	Normalization of serum and urine free copper levels *
Choice of medication	Zinc (100–200 mg daily of elemental zinc)
Monitoring treatment	Normalization of serum non-ceruloplasmin copper levels * (<100 μg/L), normalization of urinary copper (<100 μg/L)

Note: *: Serum non-ceruloplasmin copper is calculated by extracting the ceruloplasmin bound copper from the total copper. Ceruloplasmin contains 0.3 percent of serum copper.

**Table 2:** Clinical trial testing anti-copper-based therapies on cognition and Alzheimer’s disease.

Authors, year	Treatment	Sample Size	Design	Clinical Outcomes
Van Rhijn et al. 1990 [75]	Zinc-sulphate and sodium selenite	A total of 15 AD	Dietary supplementation study	Improved performance on the anomalous sentences repetition test, colored progressive matrices, graded naming test and digit copying test
Crapper McLachlan et al. 1991 [76]	Desferrioxamine mesylate (metal chelator)	A total of 48 AD	Single-blind trial	Slowing of clinical deterioration, increased activities of daily living in the treatment group
Constantinidis 1992 [71]	Zinc-hydrogenaspartate	5 presenile AD 5 senile AD	Follow-on study	Improved memory, understanding, communication and social interaction in 8 patients
Potocnik et al. 1997 [74]	Zinc-methionine	4 AD	Open-labelled pilot study (12 months)	Improved performance on the cognitive tests
Squitti et al. 2002 [77]	D-penicillamine (Copper chelator)	A total of 18 AD (treated: placebo 1:1)	Double-blind, placebo-controlled 6 months trial	No widespread benefit on cognitive outcomes; placebo patients did not worsen in the 24 weeks follow-up; peroxides in serum from patients taking D-penicillamine decreased by 29% with respect to their t0 evaluation found (F1,16 = 4·52; P = 0·049).
Ritchie et al. 2003 [78]	Clioquinol	A total of 36 probable AD (treated: placebo 1:1)	Double-blind, placebo-controlled, parallel group randomized 36 weeks study	No effect on cognitive outcomes at any of the time points assessed. Significant improvements in MMSE when the groups were stratified by their level of impairment at baseline; plasma Aβ declined in the treated group and increased in the placebo group
Maylor et al. 2006 [79]	Zinc-gluconate (Zenith)	387 healthy older adults	Randomized double-blind placebo-controlled 6 months study	Few significant benefits in visual memory, working memory, attention and reaction time were obtained using the Cambridge Automated Neuropsychological
Lannfelt et al. 2008 [80]	PBT2 (copper/zinc ionophore)	A total of 78 Early AD (treated: placebo 1:1	Phase II, double-blind, randomized, placebo-controlled 12 months trial	No significant difference in the Neuropsychiatric Test Battery (NTB); dose-dependent reduction of Aβ concentrations in the CSF and positively impacted on two executive function component tests showing significant improvement over placebo in the PBT2 250 mg group: category fluency test (2.8 words, 0.1 to 5.4; *p* = 0.041) and trail making part B (–48.0 s, –83.0 to –13.0; *p* = 0.009).
Faux et al. 2010 [81]	PBT2 (copper/zinc ionophore)	40 AD	Phase II double-blind, randomized, placebo-controlled 12 weeks trial	Improvement on NTB Composite or Executive Factor z-scores
Brewer 2012 [44]	Zinc (Adeona) 150 mg/day	A total of 42 AD mild-moderate AD patients (treated: placebo 1:1)	Phase II double-blind, randomized, placebo-controlled 12 weeks trial. Primary outcome: ADAS-Cog	No significant effect on primary clinical outcome; post hoc analyses limiting the analysis to those patients aged 70 years and older (14 zinc treated patients vs. 15 placebo patients) revealed statistically significant better cognition scores in the zinc-treated patients vs. controls in ADAS-Cog (*p* = 0.037) and CDR SOB (P ¼ 0.032), with near significant results in MMSE (*p* = 0.07)
Villemagne et al 2017 [82]	PBT2 (copper/zinc ionophore)	40 AD (12-month double-blind phase) (placebo = 15, PBT2 = 25), and 27 subjects12-month (placebo = 11, PBT2 = 16)	A randomized, exploratory molecular imaging study targeting amyloid beta with PBT2 in AD, 12-month phase in a double-blind and a 12-month open label extension phase trial design	There was no significant difference between PBT2 and controls at 12 months, likely due to the large individual variances over a relatively small number of subjects
